# Staying vigilant about the sleep–wake states—is one question the whole story?

**DOI:** 10.1093/sleepadvances/zpae024

**Published:** 2024-05-02

**Authors:** Osman S Ipsiroglu, Gerhard Klösch, Karen Spruyt

**Affiliations:** Department of Pediatrics, University of British Columbia, Vancouver, BC, Canada; Department of Pediatrics, Medical University of Vienna, Vienna, Austria; Department of Neurology, Medical University of Vienna, Vienna, Austria; NeuroDiderot, INSERM, Université Paris Cité, Paris, France

Charles Dickens, the famous British novelist, described the consequences of sleep-disordered breathing (SDB) on the daytime well-being of Joe, also known as the “fat boy,” in his novel *The Pickwick Papers* [1]. This description included how Joe’s daytime functioning impacted his emotional well-being and the social stigma associated with SDB. Dickens accomplished this feat 160 years prior to the American Academy of Sleep Medicine’s consensus statements on SDB [2]. *The Pickwick Papers* serve as a comprehensive portrayal of the sequelae, particularly the suffering associated with SDB. Dickens achieved this through his empathetic, qualitative, and descriptive *case report,* while utilizing the concepts of objective, curiosity-driven, and impartial observational skills to describe Joe’s *vigilance*. His work captured not only Joe’s struggles to stay awake and concentrated, his *functioning at daytime*, but also showcased the dimension of other individuals facing similar challenges due to disturbed sleep health. Dickens’ observations may frame the investigative journey of scientists and clinicians today.

While at the global level, WHO and other decision-making bodies have led the development of guidelines concerning topics such as physical activity, roadmaps on cancer, and the Global Diabetes Compact [3], affected functioning due to sleep disturbances has been systematically overlooked and/or portrayed as a single central theme. On the other hand, societal shifts and the fourth industrial revolution have increased worldwide public interest in sleep. Recently, the World Sleep Society has issued an urgent call to action to integrate sleep into global (daytime) public health policy to address various health concerns, including cardiometabolic disorders, impaired immune responses, overall productivity, dementia, child development, and mental health [4]. This poses the questions: *How can we increase awareness of the commonality of our sleep–wake or wake–sleep states and what are the measures to detect disturbances or even disorders? How can we screen for sleep health using only one question?*

Sleep is a complex physiological function. The processes of sleep are still not fully understood and are the subject of ongoing research, continually relativizing the value of our “gold standards” with each investigation. For example, the local and asynchronous distribution of slow waves across the cortical surface, which reflects the flexibility of our physiological sleep needs [5], is not taken into account by conventional sleep classification techniques [6]. Phenomena like first night effect [7] or sociocultural norms may interfere or undermine the objectiveness of lab-based sleep studies [8], and the mismatch between objective and subjective sleep perceptions [9, 10] inform the current norms.

Death and the fear of death during sleep have prejudiced perceptions of sleep [11]. Thus, also the diagnostic and treatment approach to poor sleep in childhood has been a risky endeavor. The field of contemporary pediatric sleep medicine’s roots can be linked to investigations into sudden infant death syndrome during the 1970s and 1980s, as outlined by Kahn [12]. The key questions during this research were *what is the norm*, *what exceeds the norm,* and *how this might lead to (sudden infant) death*. Historically, in ancient Greek mythology, sleep *(Hypnos)* and death *(Thanatos)* were twin brothers [13], and nighttime prayer traditions were initiated due to a fear of death during sleep [11]. Such narratives long played a role in framing our perception of the concept of sleep. On the contrary, science-oriented thinking and changes in cultural norms have coined the focus on quantitative measures of sleep. It is a fact that social, psychological, and biological factors influence physiological measures, but from the individual’s perspective, all is seen and perceived within their cultural framework [9]. The controversial discussion on co-sleeping practices with children reflects this paradigm change [14].

Mounting evidence suggests that chronic poor sleep, impacting emotional, cognitive, and physical well-being, may stem from a variety of factors such as poor sleep habits, mental health, threatening life events, or medical conditions. It also aggravates the clinical presentation, particularly in the case of developmental or genetic disorders. In childhood, moreover, the underlying pathophysiological targets for treating specific sleep disturbances remain widely unknown, except for biochemical imbalances, which should already be screened in the community at the public health level [15–17]. This lack of clarity and the variety of applied methodologies lead to controversial results. Our desire to treat and achieve tangible success often prompts us to explore experimental approaches that are based on weak evidence, such as the use of high doses of melatonin to treat any form of poor sleep [18–20]. The harsh reality in pediatric sleep medicine is that we predominantly use off-label drugs [21].

Attention deficit hyperactivity disorder serving as an example of the interrelation between sleep and daytime behaviors reflects a dimension of this sleep–wake state complexity [22]. Although leading pediatric or childhood psychiatric associations suggest treating sleep disturbances before diagnosing attention deficit hyperactivity disorder, doing so presents challenges [23–26]. This is likely due to the lack of a clinical roadmap or protocol for primary screening and assessment at the community level. Thus, given the notion that optimal long-term daytime functioning is existential, the majority of therapeutic interventions target daytime symptoms. This aggravates the challenge of how to screen for sleep health using only one question, but this challenge may also point the way.

Here, the idea of vigilance, the ability of an individual to respond appropriately to environmental stimuli in order to ensure survival, serves as a synopsis of daytime functioning. The innovative use of this concept, introduced by British neurologist Henry Head 100 years ago in 1923 [27], provides valuable insights into wake behaviors [28]. In the animal kingdom, reduced vigilance leads to being eaten by predators or hunters. In 21st century humans, affected functioning, due to both impaired vigilance and inappropriate responses to environmental stimuli, are of concern. Namely, we often seek to correct such malfunctioning or affected vigilance promptly and effectively through compensatory coping, such as the excessive use of caffeine and/or other stimulants. While questions related to daytime well-being or quality of life are unspecific and may not necessarily reflect insufficient sleep [29] and while functionality can vary based on developmental state, intellectual abilities, and cultural norms, the concept of vigilance serves as a neutral descriptor for the ability to “survive the day.” Obviously, poor vigilance can be caused by multiple factors, but a majority of them are associated with underlying and/or overlooked poor sleep [9, 29]. Therefore, we hypothesize the concept of vigilance to be more specific for exploring and capturing affected sleep health, whether caused by sleep disturbances and/or disorders [28]. Such neutral exploratory questioning exceeds the culturally coined notions of the individuals involved in this discussion (including professionals with different training backgrounds) and it also answers the question of whether we should focus on the sleep state or the awake state, as vigilance allows a transition to both states. In summary, vigilance presents an exceptional opportunity for sleep medicine to excel!

If our arguments have not persuaded you that vigilance is the “starter’ which should be used for screening of factors affecting sleep health, let us step back and again revisit Dickens’ *The Pickwick Papers*. Dickens” conceptual understanding is rooted in empathy, enabling his exploratory observations of Joe’s vigilance, including affected daytime-functioning, quality-of-life, and well-being in context with his sleep health. In the professional setting, the concept of vigilance promotes empathy and encourages transdisciplinary thinking, ensuring that the fundamental public health principles are followed and that basic factors, such as sleep health measures associated with lifestyle, are not overlooked [9]. Furthermore, vigilance will encourage a step-by-step approach, as promoted by public health experts who aim to establish prevention concepts. In consequence, before a referral to a child and adolescent psychiatrist is made, the community practitioner may consider sleep health, in Joe’s case SDB, as a differential diagnostic consideration. The concept of vigilance will also allow each discipline to go down its own path while being mindful of an overall roadmap of stepped care. This is important as, in the Western-centric world, recognition of the value of sleep on emotional and cognitive development, functioning, and well-being is relatively recent. Vigilance not only allows a bridge between modern, Western-centric beliefs and traditional cultural concepts, but also brings in a new perspective for evaluating behaviors labeled as “disruptive” in children or as “challenging” or “worrisome” in adults in context with affected sleep health. This innovative perspective enables a more impartial and objective roadmap, along with the opportunity to reframe disruptive behaviors through the lens of sleep health. It is important to recognize that “norms” are shaped by the narratives of the storyteller [30–33]. And so, as the narrative progresses, standing at this intersection in the field of sleep, the “via vigilance” united sleep–wake state concept marks an essential first step in patient-professional communication that avoids training bias. Stay vigilant!

## Data Availability

Not applicable.
